# Impact of Early-Age Curing and Environmental Conditions on Shrinkage and Microcracking in Concrete

**DOI:** 10.3390/ma18133185

**Published:** 2025-07-05

**Authors:** Magdalena Bacharz, Kamil Bacharz, Wiesław Trąmpczyński

**Affiliations:** 1 Department of Material Strength and Diagnostics of Structures, Faculty of Civil Engineering and Architecture, Kielce University of Technology, 25-314 Kielce, Poland; 2 Department of Building Structures, Faculty of Civil Engineering and Architecture, Kielce University of Technology, 25-314 Kielce, Poland; wtramp@tu.kielce.pl

**Keywords:** non-loaded/unloaded concrete, shrinkage, concrete shrinkage estimation, acoustic emission, NDT, new diagnostic method, early-age damage, standards, EN 1992-1-1:2023, maturation

## Abstract

This study analyzed the effects of curing and maturation on the formation of shrinkage strain and destructive processes in concrete. Experimental tests were performed on commonly used concrete, class C30/37, with basalt aggregate and blast furnace cement tested: at constant temperature after water curing, at constant temperature without water curing, and under cyclically changing temperature without prior curing. Shrinkage strain was measured for 46 days with an extensometer on 150 × 150 × 600 mm specimens, and the acoustic emission (AE) method was used to monitor microcracks and processes in concrete in real time. The results were compared with the model according to EN 1992-1-1:2023. It was found that this model correctly estimates shrinkage strain for wet-curing concrete, but there are discrepancies for air-dried concrete, regardless of temperature and moisture conditions (constant/variable). Correlation coefficients between shrinkage strain increments and process increments in early-age concrete are proposed. Correlations between shrinkage strain and destructive processes occurring in concrete were confirmed. It was found that by using correlation coefficients, it is possible to estimate internal damage in relation to shrinkage strain. The results indicate the need to develop guidelines for estimating shrinkage strain in non-model environmental conditions and demonstrate the usefulness of the nondestructive AE method in diagnosing early damage, especially in concrete structures exposed to adverse service conditions.

## 1. Introduction

The shrinkage of concrete in the early stage of maturation is one of the key phenomena affecting the durability and quality of a structure. This process occurs in the first few hours and days after the concrete mix is made and can lead to crack formation and a decrease in the strength of the structure.

Concrete shrinkage is a change in the volume of the concrete mixture and hardening concrete that occurs due to the hydration process and loss of water [1,2]. We can distinguish the following types of shrinkage: plastic shrinkage, chemical shrinkage, autogenous shrinkage, shrinkage due to drying, thermal shrinkage, and carbonation shrinkage. The shrinkage of the concrete depends on many factors, including the composition of the concrete mixture, that is, not only the type of aggregate [3,4], cement [5] but also admixtures [6] and additives [7], including fibers such as steel or polypropylene [8,9,10], the age of the concrete (especially sensitive to lack of curing is young concrete up to 7 days [11]), the technology of its execution, and temperature and humidity [12,13,14]. The latter two parameters in particular have a strong influence on ensuring the durability of many structures, such as massive concrete structures [15] or airport pavements [16]. Higher temperature accelerates water evaporation, which can result in higher shrinkage strain, and in the case of low temperatures, the water locked in the air pores freezes, leading to volume changes and internal stresses [1,2,12]. This is especially important for early-age concrete that has not yet reached adequate tensile strength, which can affect the development of microcracks and the durability of the concrete.

Many papers provide an overview of the concept of concrete shrinkage taking into account the causes of its occurrence [17], the mechanisms that cause it [18], and strategies for mitigating its negative effects, i.e., microcracking [19].

Due to the different mechanisms that cause each type of shrinkage, a comprehensive modeling of it would require a comprehensive analysis of the concrete microstructure and physicochemical processes, which is difficult to do in practice.

An important issue was described by the authors of [20], who proposed an approach to predict non-stationary moisture transport in porous materials (including concrete). Therefore, the implementation of this method to identify areas prone to shrinkage and microcracking in unloaded concrete could be useful from a material sustainability and quality point of view.

Estimation of shrinkage strain is important in engineering practice at various stages of structure life, from design through use, to damage diagnostics. Many models are being developed on the basis of which shrinkage deformations can be estimated in concrete, including those described in [20,21,22,23,24] and the guidelines implemented in construction standards. Especially the latter require continuous validation and updating to include new materials and technologies (e.g., high-performance concretes [25], mineral additives [26,27], 3DPC (in 3D-printed concrete) [28]). Moreover, the results obtained using empirical formulas included in the standards may differ from the experimental values, among other reasons due to the overlap of many factors during concrete curing, e.g., in extreme conditions, which cannot be predicted or modeled. Hence, it is very important to validate standard approaches based on experimental data, thanks to which standard provisions are constantly improved and updated, as was the case with the European standard related to construction, Eurocode 2: Design of concrete structures (EN 1992-1-1:2023) [24], the new version of which was implemented in Poland in 2024 as PN-EN 1992-1-1:2024 [29].

Therefore, shrinkage studies are still relevant, among other reasons because of the negative effects that can occur in practice. As a result of various shrinkage strain, internal stresses occur in the material (usually tensile stresses of the external section and compressive stresses of the internal section [30,31], and in places of inhomogeneity (for example, at the cement paste–aggregate interface, local stresses occur), which can be divided into “macro,” “micro,” and “submicro” stresses. If their value exceeds the tensile strength of concrete, microcracks appear in places of stress concentration. They can be at the site of crack initiation, their propagation, and cause failures already at an early stage of use, an example of which can be problems caused by shrinkage both in prefabrication plants [32] and in elements of structures in use, including: industrial floors [33], parking lots [34], tanks [35], retaining walls [36], foundations of nuclear power plants [37], or spans of bridge structures [38].

It is therefore important to predict the value of shrinkage strain, as well as its effects. For defects that appear on the surface of a single element, two-image photogrammetry, such as the ARAMIS system, can be used to analyze them [39]. On the other hand, in the case of structures that require continuous monitoring to detect an emergency condition, sensors that record the real-time operation of the structure are used. In recent years, there has been a development of methods based on machine learning algorithms [40,41], which greatly facilitate the processing of a large quantity of data in terms of classification and detection of anomalies that may indicate an emergency condition. An example of this is papers [42,43], whose authors proposed methods to predict in real time the path of concrete cracks, allowing damage assessment based on the collected data. Such approaches may be an effective alternative to traditional damage prediction methods, which may not be sufficient for unloaded concrete.

The use of input data, such as material characteristics or load history, makes it possible to identify potential damage zones, even before they occur. Similar solutions are also presented by other studies, such as damage detection using acoustic emission [44,45,46]. An example of this is the IADP (identification of active destructive processes) method, one of the nondestructive methods [47,48] developed for loaded [49,50] and no loaded concrete in order to detect invisible microcracks arising in real time.

Previous diagnostic approaches based on acoustic emission have focused mainly on the analysis of AE signals in the context of localization of emission sources or classification of fracture mechanisms. However, attempts have rarely been made to directly relate the increase in AE signals to the actual deformation state of the material and the destructive processes that occur, especially in non-loaded concrete. This study fills this gap by looking for the relationship between AE signals and the course of shrinkage. This makes it possible not only to detect but also to predict areas potentially at risk of material degradation at an early stage of the material’s service life.

In this paper, the results of experimental tests of shrinkage strain measured through 46 days on cuboid specimens of commonly used concrete, matured in varying temperature and humidity conditions with or without wet curing, were compared with the shrinkage strain prediction model according to EN 1992-1-1:2023 [24]. Furthermore, the acoustic emission (AE) method—IADP (identification of active destructive processes)—which allows the identification and localization of damage mechanisms accompanying shrinkage and has been used repeatedly, successfully by the authors [51,52], was also used to monitor microcracks and processes in concrete in real time. Correlation coefficients between shrinkage strain increments and process increments in early-age concrete are proposed. Concrete samples of class C30/37 with basalt aggregate and blast furnace cement at constant temperature after water curing, at constant temperature without water curing, and under cyclically changing temperature without prior curing in water were tested. The shrinkage strain was measured for 46 days with an extensometer in 150 × 150 × 600 mm specimens.

This study was aimed at supplementing the results of the tests described in [52] with elements matured under cyclic temperature conditions without curing in water after removal from the molds, supplementing the analyses of shrinkage deformation by comparing the experimental results with the new version of the standard Eurokode 2, as well as verifying the correlations from the paper [52] and the adopted coefficients. The results are discussed and elaborated.

## 2. Materials and Methods

### 2.1. Test Elements

The tests were carried out on 3 series (C1–C3) of commonly used concrete made with basalt aggregate from the Gracze quarry located in the Opole woivodeship, Poland and CEM III/A 42.5N-LH/HSR/NA blast furnace cement from Górażdże cement plant located in Chorula, in the Opole woivodeship. The composition of the concrete mix per 1 m^3^ included: 581 kg of basalt aggregate fraction 2–8, 731 kg of basalt aggregate fraction 8–16, 360 kg of cement, 691 kg of river sand, and 180 kg of tap water. The performance characteristics of the cement (based on [53]) are summarized in Table 1.

The main properties of the basalt aggregate (based on [54,55]) are summarized in Table 2.

Three cuboid samples of dimensions 150 × 150 × 600 mm from each of the concrete series C1–C3 were used. After removal from the mold, sample C1 was cured in water (humidity 100%) for 10 days. The remaining samples were tested without prior curing. The parameters of the concrete mixtures are shown in Table 3.

**Figure 1:**
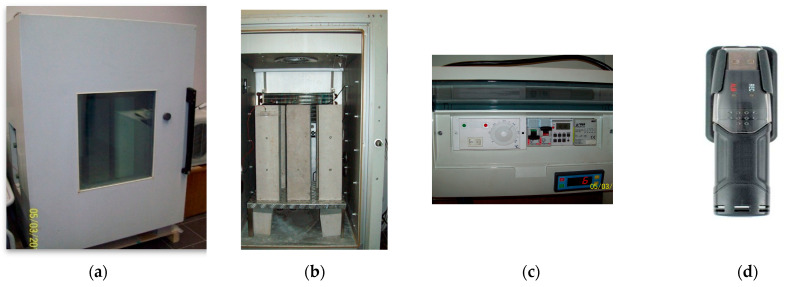
Thermal test chamber setup: (**a**,**b**) the thermal chamber; (**c**) thermostat; (**d**) temperature and humidity sensor.

Samples C1 and C2 matured at a constant temperature of 22 °C, while samples C3 matured at a cyclically variable temperature in 12 h periods of heating to +42 °C and then cooling to −5 °C (Figure 1). The adopted temperature range is a typical design range of temperature changes resulting from local climatic conditions. It makes it possible to evaluate the behavior of the structure under daily use.

**Figure 2:**
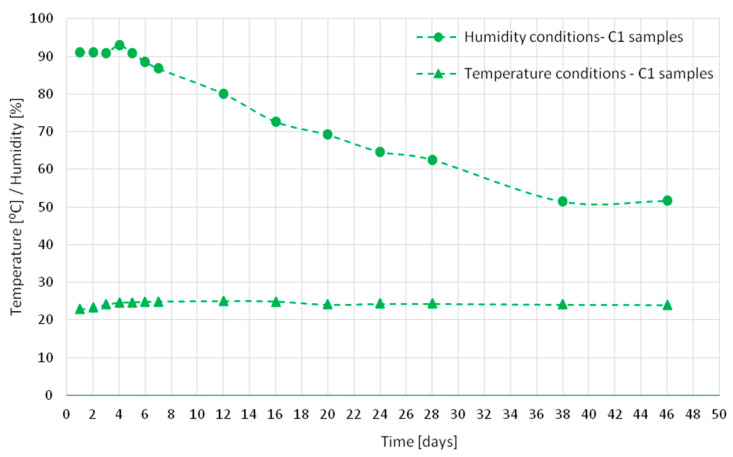
Humidity and temperature conditions for series C1.

**Figure 3:**
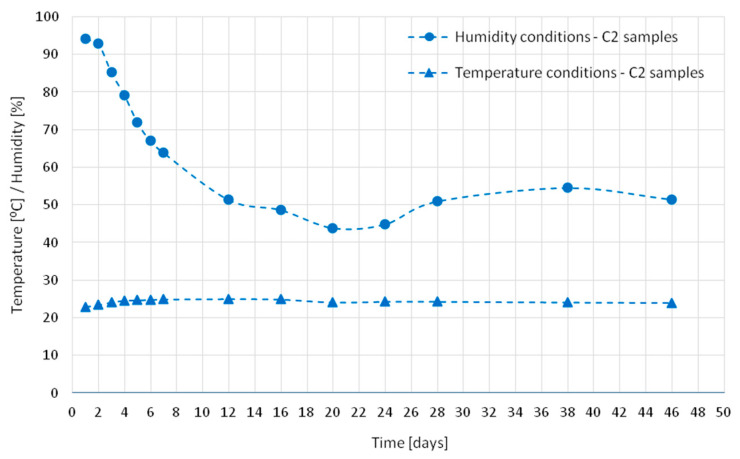
Humidity and temperature conditions for series C2.

**Figure 4:**
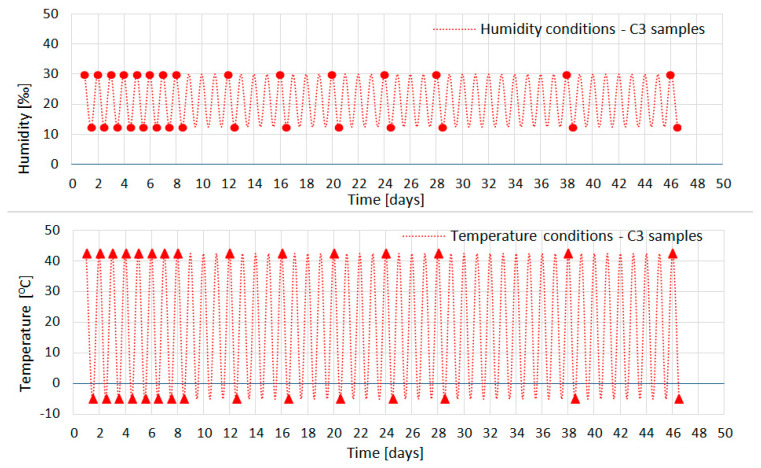
Humidity and temperature conditions in the thermal chamber for series C3.

Characteristics of all samples matured in a chamber in ambient temperature and humidity were recorded. Figure 2, Figure 3 and Figure 4 show the temperature and humidity recorded in the chamber during the tests of concrete series C1 (Figure 2), C2 (Figure 3), and C3 (Figure 4). The circular and triangular markers on the humidity and temperature graphs, respectively, indicate the points at which strain measurements were taken and the 12 h measurement of the acoustic emission signal prior to a given day was completed. To perform the measurements, the samples were taken out of the chamber and taken to a technical room, where the measurement lasted approximately 1 h in total. The samples were placed in the chamber and the temperature inside was set at 22 °C for samples C1 and C2 and −5 °C or +42 °C, respectively, depending on the cycle that was to follow the test (heating or cooling) for sample C3. The humidity accompanying the tests inside the chamber was recorded, but not imposed due to the need to turn off/silence all noise-emitting devices inside the chamber. For this reason, the cooling device was located outside the insulated chamber (Figure 1).

### 2.2. Research Methods

#### 2.2.1. Strain Measurements and Prediction

**Figure 5:**
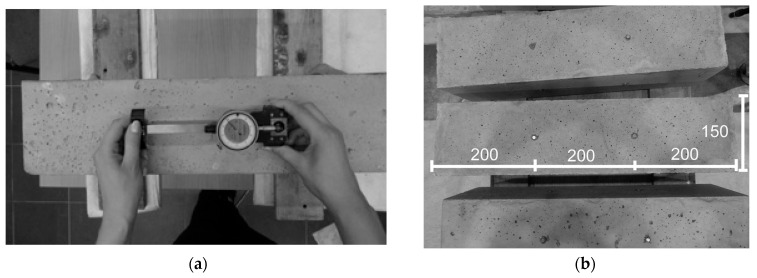
Test specimens: (**a**) measurement with an extensometer; (**b**) distribution of measurement points in [mm].

The shrinkage strain was measured on 4 sides of each sample using an 8-inch extensometer, shown in Figure 5a. The spacing of the metal sensors on the wall of the rectangular sample is shown in Figure 5b and was 20 cm.

The experimentally obtained shrinkage strain was compared with the results estimated according to EN 1992-1-1:2023 [24]. Figure 5 shows a simplified scheme for estimating shrinkage strain according to EN 1992-1-1:2023.

#### 2.2.2. Acoustic Emission Signal (Destructive Process) Measurements

**Figure 6:**
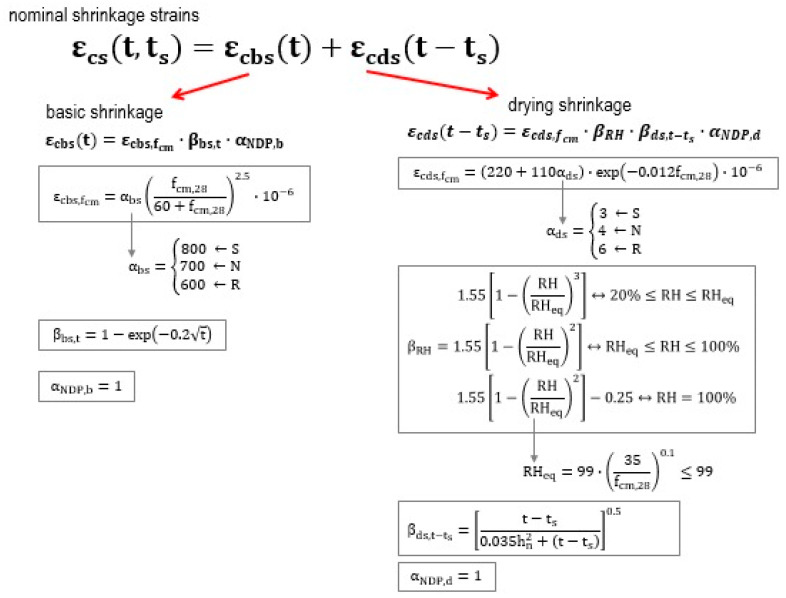
Scheme to estimate shrinkage strain according to EN 1992-1-1:2023.

Acoustic emission tests were performed on all concrete samples from the C1–C3 series. On one wall of each of the 9 samples, 2 piezoelectric sensors were connected 3 cm from the top and bottom edge of the sample along the wall axis, as shown in Figure 6.

In the study, VS-30V sensors were used to record signals, which convert active phenomena occurring in concrete into electrical signals. This type of sensor, with a measurement range of 25–80 kHz, allows the detection of low-frequency signals corresponding to phenomena occurring in non-loaded concrete. Additionally, to improve the contact between the sensor and the concrete and to avoid excessive energy losses at the contact between the sensor and the concrete surface, a coupling agent was used in the form of silicone paste and elastic tape. A PC with Mistras-2001 software was used to record signals. In addition, the measurement system itself was supplemented by preamplifiers with AE signal gain ranges of 20, 40, and 60 decibels, which additionally ensured, at a threshold of 30 dB, that the signals generated could be recorded despite the attenuation present in the concrete.

The results were analyzed using the Noesis program. Acoustic signals recorded over a period of 46 days were assigned to 3 standard classes of destructive processes, according to the IADP (identification of active destructive processes) method [50,51,56].

The database of reference signals includes the following.

1. Class 1 signals indicate microcracks in the cement paste and in contact between the paste surface and aggregate grains.
2. Class 2 signals indicate the development of internal microcracks.
3. Class 3 signals indicate the formation of microcracks on the concrete surface.

**Figure 7:**
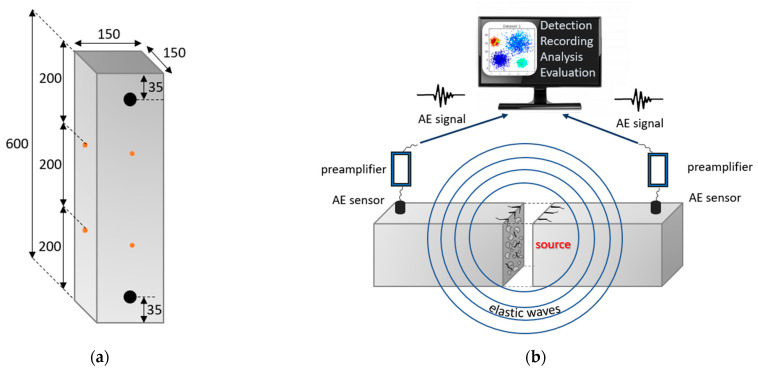
(**a**) Location of AE sensors (black) and metal benchmarks (orange) to measure the strain on the sample (in millimeters); (**b**) scheme of concrete testing using the acoustic emission method.

The test scheme is shown in Figure 7b.

## 3. Results

### 3.1. Shrinkage Strain

#### 3.1.1. Experimental Results—Strain

Figure 7 shows the average shrinkage strain measured in series C1, C2, and C3 samples. Points on the graph indicate the days on which the strain were measured with the extensometer. The results of each series differed from each other. After 46 days of curing, the highest strain value of ~0.7% was recorded in the concrete of series C3, which was cured under cyclic temperature conditions (without curing). Lower levels of strain were observed in samples cured under conditions of a constant temperature of 22 °C: ~0.5% in concrete samples of series C2, which was not subjected to curing in water after removing samples from the molds, and the lowest ~0.3% in samples of series C1 cured in water for 10 days before the test.

The results of C1 and C2 were already presented in [52]. In this paper, they are reworked and discussed together with data obtained for C3 samples. This allows the drawing of new conclusions.

**Figure 8:**
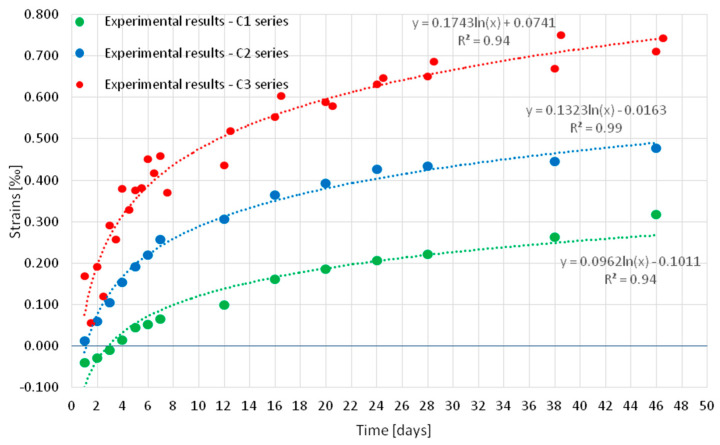
Results of strain tests of specimens from series C1, C2, and C3 with trend lines.

For the strain values, trend lines were developed along with determination coefficients (R2, Figure 8), which for logarithmic functions were close to one. This showed that the logarithmic function accurately matched the strain values.

A high level of fit of the strain to logarithmic functions was observed in each series of concrete (C1 to C3).

#### 3.1.2. Shrinkage Strain Predicted by the EN 1992-1-1:2023 Standard

**Figure 9:**
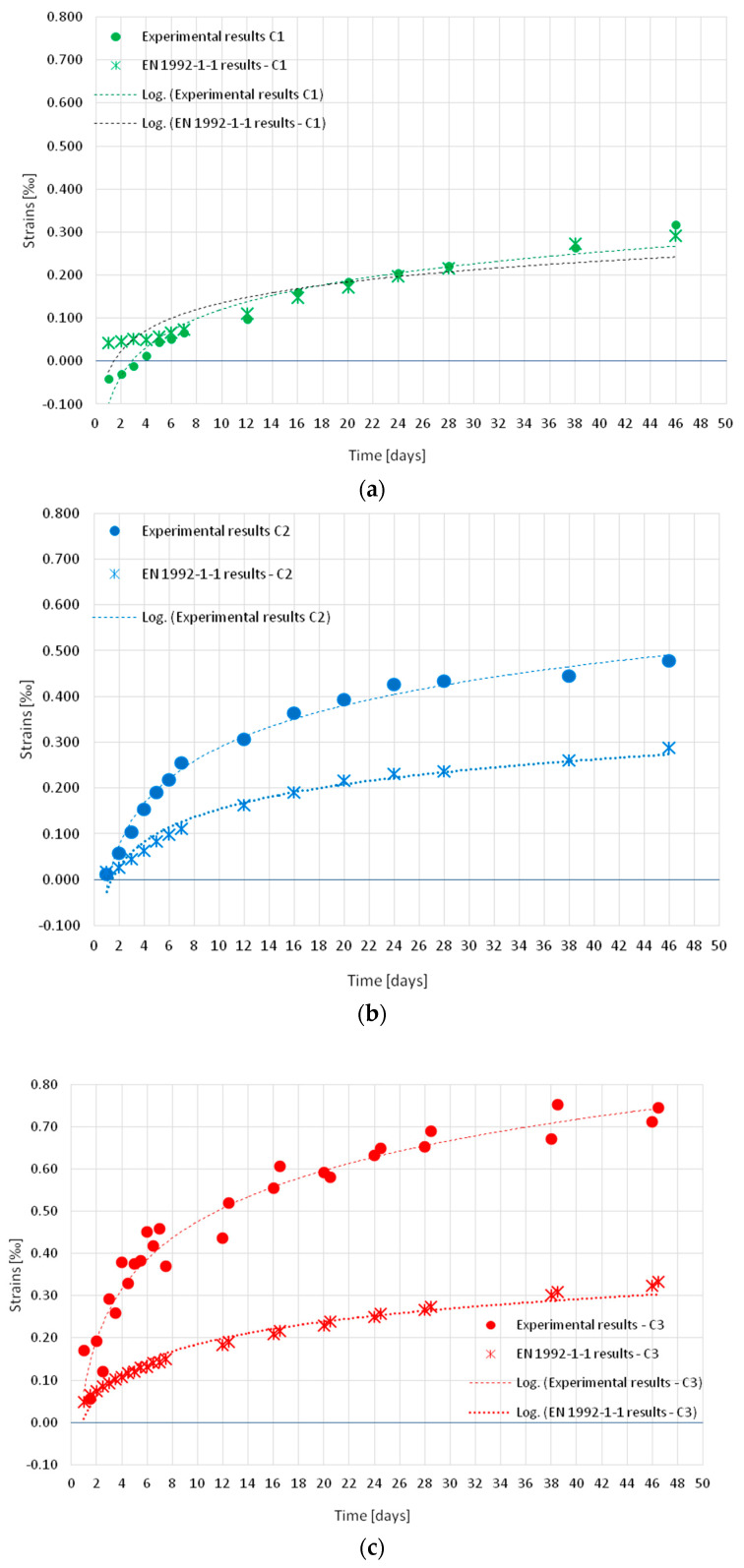
Strain results Graph of strain versus time in the samples: (**a**) C1; (**b**) C2; (**c**) C3.

The first analysis was to compare the strain obtained experimentally with the values estimated according to EN 1992-1-1:2023. The results of the analyses are summarized in Figure 9a–c for each concrete series: C1, C2, and C3.

**Figure 10:**
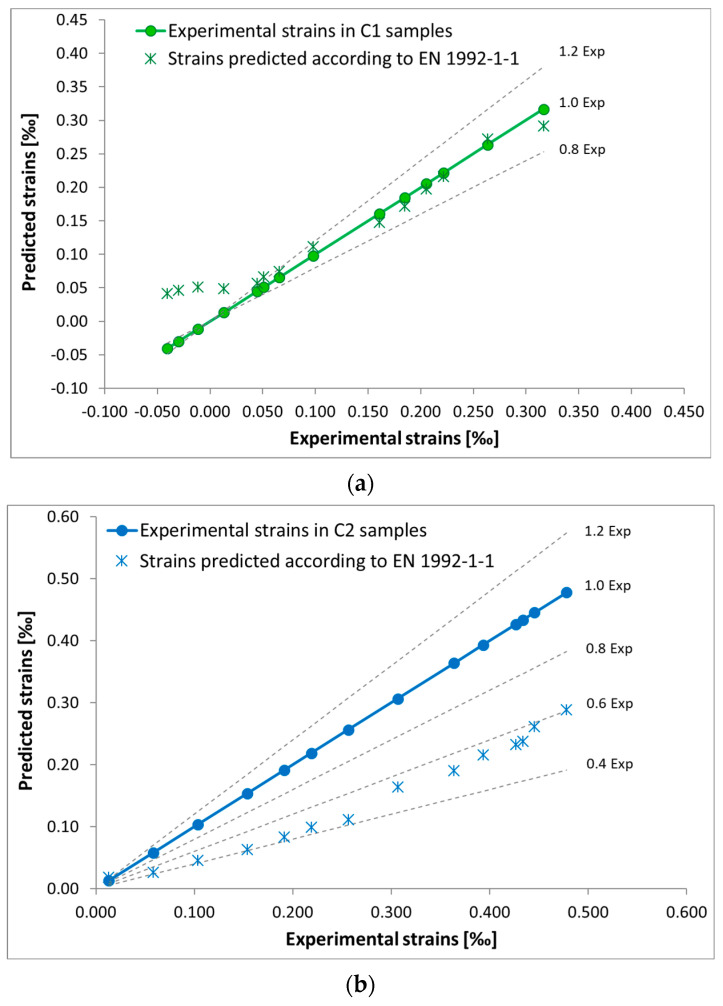

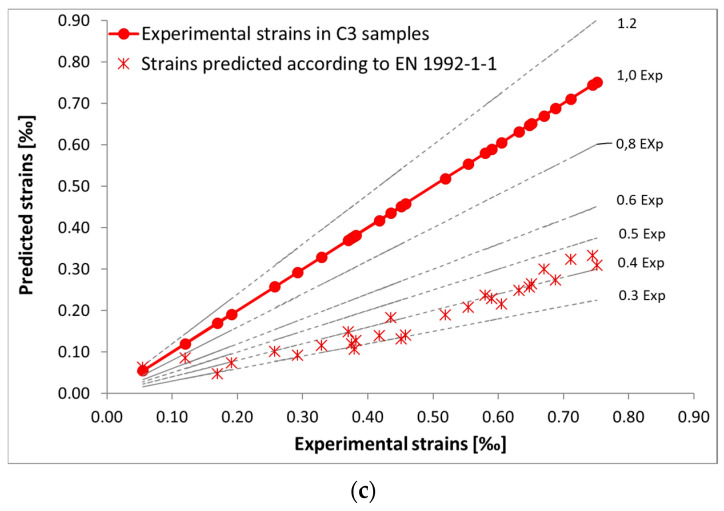
Strain results in the samples: (**a**) C1; (**b**) C2; (**c**) C3.

Figure 10 shows the correlations between experimental and predicted strain according to EN 1992-1-1:2023, with concrete series accompanied by error levels.

In the case of C1 (Figure 9a and Figure 10a), the experimental and estimated results according to EN 1992-1-1:2023 coincided, except for the initial stage of the test, in which swelling was observed in the C1 specimens.

In the case of C2 concrete, which was not cured in water after being removed from the molds (Figure 9b and Figure 10b), the results estimated according to EN 1992-1-1:2023 differed from the experimental values and the underestimation was up to 60%.

In the case of C3, which was not subjected to curing in water and was matured under cyclically variable conditions (Figure 9c and Figure 10c), the results estimated according to EN 1992-1-1:2023 underestimated the experimental values by up to 65%–70%.

Based on the results obtained, it can be stated that the strain estimated for C1 (Figure 9a and Figure 10a) was within ±20% deviation from the values obtained experimentally. In the case of concrete not subjected to curing in water after removal from the molds, C2 (Figure 9b and Figure 10b), cured at a constant temperature of +22 °C, and C3 (Figure 9c and Figure 10c) significantly differed from the values obtained experimentally. Furthermore, the greatest difference in results was obtained in the case of C3 after removal from molds hardened in variable cyclic temperature from −5 °C to +42 °C.

For the concrete discussed above, when there is a risk of inadequate or no cure after removing from the predicted shrinkage strain molds, an increase in the values (according to [24]) shrinkage strain of about 65% should be considered.

### 3.2. Acoustic Emission Signal Analysis

#### 3.2.1. AE Signal Analysis

**Figure 11:**
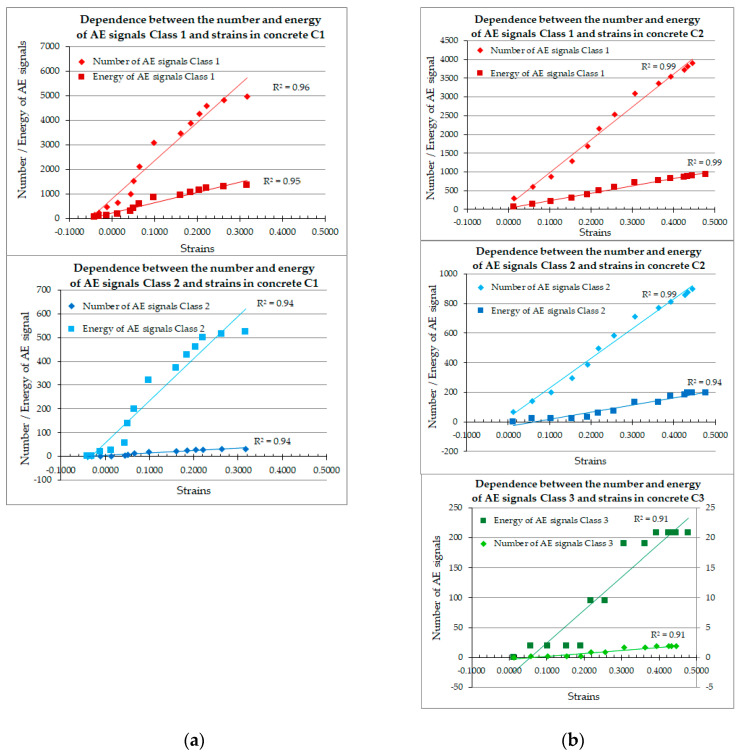
Dependence between strain, the number of destructive processes, and the energy of these processes recorded in concrete samples: (**a**) C1; (**b**) C2 and C3.

**Figure 12:**
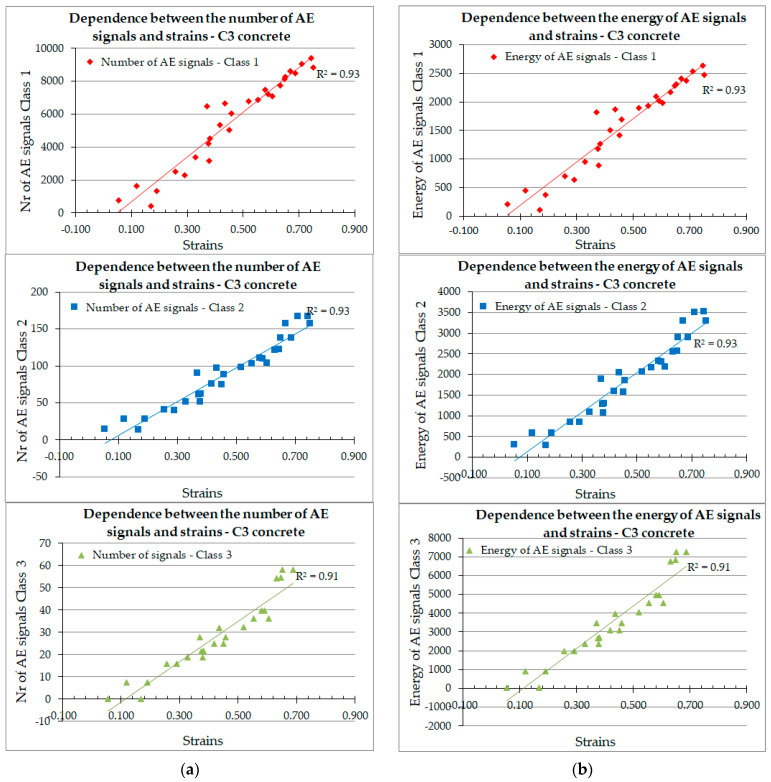
Dependence between strain and: (**a**) the number of destructive processes of classes 1, 2 and 3; (**b**) the energy of destructive processes of classes 1, 2 and 3.

The experimental results of the C1–C3 concrete shrinkage strain were correlated with the values obtained for the number of destructive processes 1–3 and the energy of these processes, obtaining a high degree of matching, as shown in Figure 11 (C1–C2 series of concrete) and Figure 12 (C3 series).

In the C1 concrete, two classes of destructive processes were recorded (Figure 11a), while in the C2 concrete, three classes of signals were recorded (Figure 11b), also accompanied by the appearance of microcracks on the concrete surface.

An analogous high degree of matching of results was obtained for concrete samples, as shown in Figure 12.

In C3 concrete, three classes of destructive processes were recorded (Figure 12), accompanied by the appearance of microcracks on the concrete surface as well.

The energy and number of AE signals recorded in concrete samples of C3 were compared with the increase in shrinkage strain. The average unit energies of AE signals of classes 1, 2, and 3 in C3-series concrete are summarized in Table 4.

#### 3.2.2. Acoustic Emission–Strain Correlation

The values obtained for shrinkage strain and destructive processes showed a very strong correlation (Figure 11 and Figure 12) in both C1 concrete (cured) and matured at a constant temperature of 22 °C, and in non-cured C2 (matured at a constant temperature) and C3 (matured at a temperature cyclically varying from −5 °C to +42 °C). For both the increase in shrinkage strain over time (Figure 8 and Figure 9) and destructive processes, a trend line [52] can be determined, which is the logarithmic function (1):

(1) $$y=A\cdot \ln\left(x\right)+B$$

It should be noted that the adoption of a logarithmic function as the trend line was considered optimal after considering several options. For the logarithmic function, the coefficient of determination R2 was high, 95% and above, and the number of coefficients that would need to be compared and determined to determine the formula (allowing the estimation of the number of signals based on shrinkage deformations) was the lowest. Using polynomial functions of higher orders, it was possible to obtain higher values of R2 coefficients due to their greater ability to match the analyzed data, while the number of coefficients that would have to be included in the estimation would be much more complicated. On the other hand, one of the authors’ goals, in addition to indicating the function itself to determine the number of signals based on shrinkage deformations, was also to optimize this solution in terms of its level of complexity. We were looking for a solution that would be applicable in engineering practice, and such a solution, in addition to an appropriate level of matching, should also be characterized by availability in terms of computational complications. In addition, it should be taken into account that the high level of matching, which characterizes the polynomial function, unfortunately constitutes its lack of usefulness in this case. This is due to the fact that acoustic emission signals can be characterized by local peaks and disturbances due to external noise not resulting from the analyzed process, which in this case were the signals associated with shrinkage, and functions with a high level of fitting are extremely sensitive to such distortions of the overall trend, which can significantly affect the entire analysis negatively. As a result of its resistance to such distortions, the logarithmic function was again found to be more favorable, showing both a high degree of matching for both AE signal growth and shrinkage deformation, while also being insensitive to distortions that arose.

Based on the similarity of the distributions of the two processes, the calibration coefficients α and β (2) were determined, thus making it possible to predict the increase in the number of AE signals from the increase in shrinkage strain obtained experimentally:

(2) $$\alpha =\frac{A_{AE}}{A_{SK}},\beta =\frac{B_{AE}}{B_{SK}}$$

where A_AE_—coefficient A of the logarithmic trend function for AE signal increment, B_AE_—coefficient B of the logarithmic trend function for AE signal increment, A_SK_—coefficient A of the logarithmic trend function for the increase in shrinkage strain, and B_SK_—coefficient B of the logarithmic trend function for the increase in shrinkage strain

**Figure 13:**
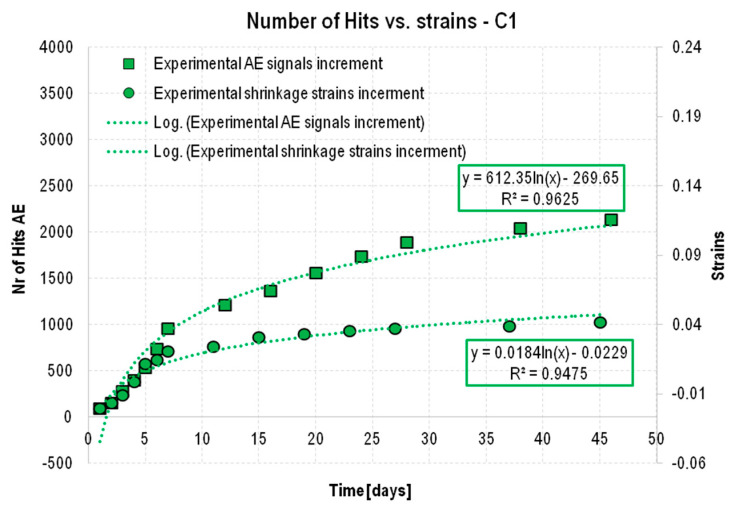
Increments in shrinkage strain and the number of AE signals recorded during the test, accompanied by assigned logarithmic trend lines.

The first step was to determine the trend-line functions for the shrinkage strain increments that were obtained experimentally and the AE signals for C1 concrete (Figure 13). Since the AE signals were recorded at 12 h intervals on days 1–7, 12, 16, 20, 24, 28, 36 and 46, the shrinkage strain increments from the same time points were analyzed.

**Figure 14:**
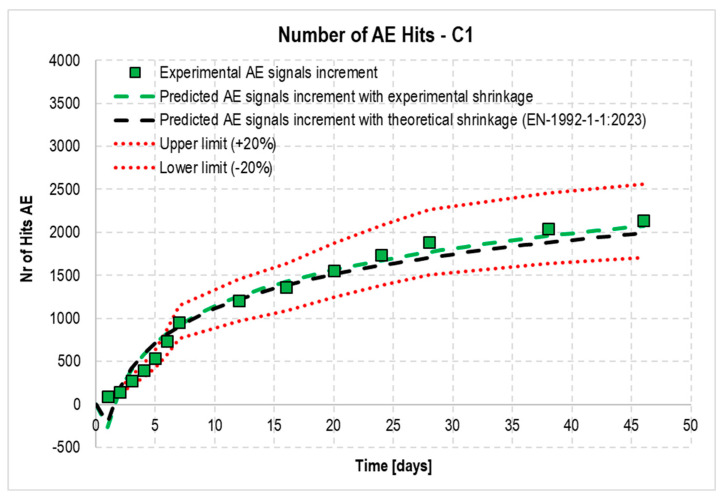
Increments in AE signals recorded during testing of C1 concrete along with theoretical increments estimated from experiments (green dashed line) or calculated based on EN 1992-1-1:2023 (black dashed line) shrinkage strain.

Based on the data shown in Figure 13, coefficients α and β were determined: α = 33,280 and β = 11,775. Next, it was verified that—taking the logarithmic trend line for the increase in the experimentally obtained shrinkage strain y = 0.184ln(x) – 0.0229 as the base function and multiplying the coefficients A and B of this function by the coefficients α and β, respectively, according to Formula (3)—the theoretical increase in the number of signals was consistent with that obtained experimentally. The results of the analysis are shown in Figure 14, where the black dashed line representing the estimated AE signal increments almost coincides with the strain values obtained experimentally and is within the assumed error limits of ±20%.

(3) $$y=\alpha \cdot A\cdot \ln\left(x\right)+\beta \cdot B$$

An analogous analysis was performed using the shrinkage strain predicted for C1 concrete according to Eurocode 2 to calculate the dependence. Similar values were obtained, and are shown with a blue dashed line in Figure 14.

**Figure 15:**
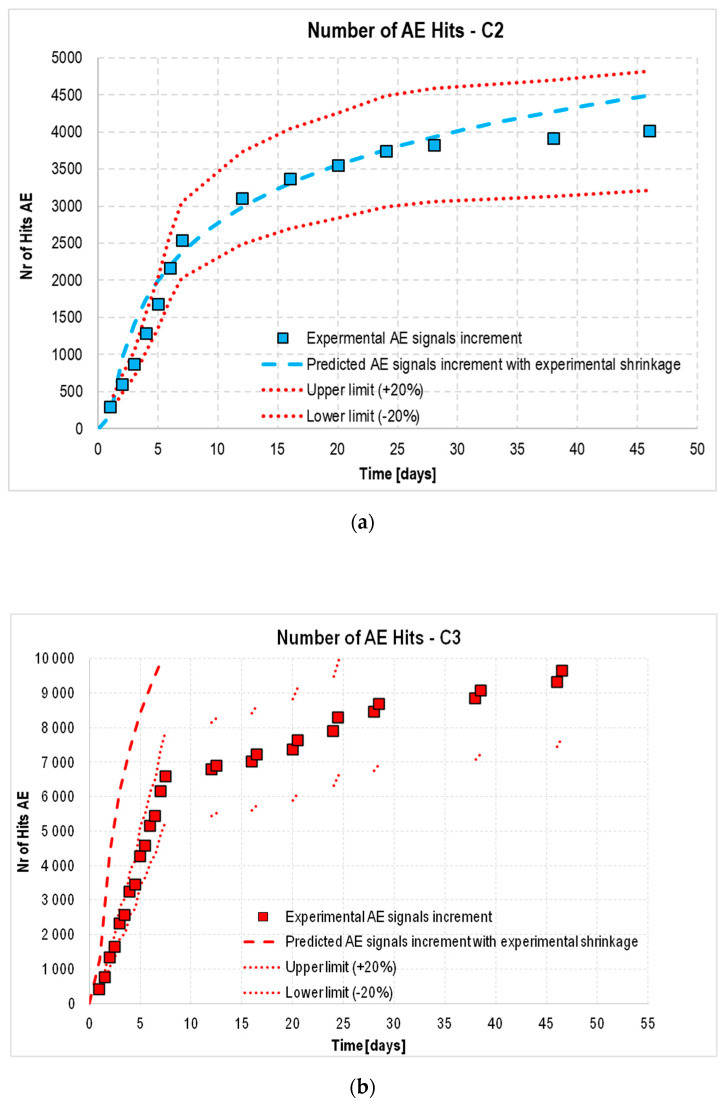
Increments in AE signals recorded during tests of concrete (**a**) C2 and (**b**) C3 with theoretical increments estimated from experimental (blue and red dashed lines) shrinkage strain.

Next, it was checked whether it were possible to use the relationship developed for C1 concrete for the other concretes that were not cured in water after demolding: C2 (matured at a constant temperature of 22 °C) and C3 (matured at a temperature that varied cyclically from −5 to +42 °C). The resulting dependences are shown in Figure 15a,b.

**Figure 16:**
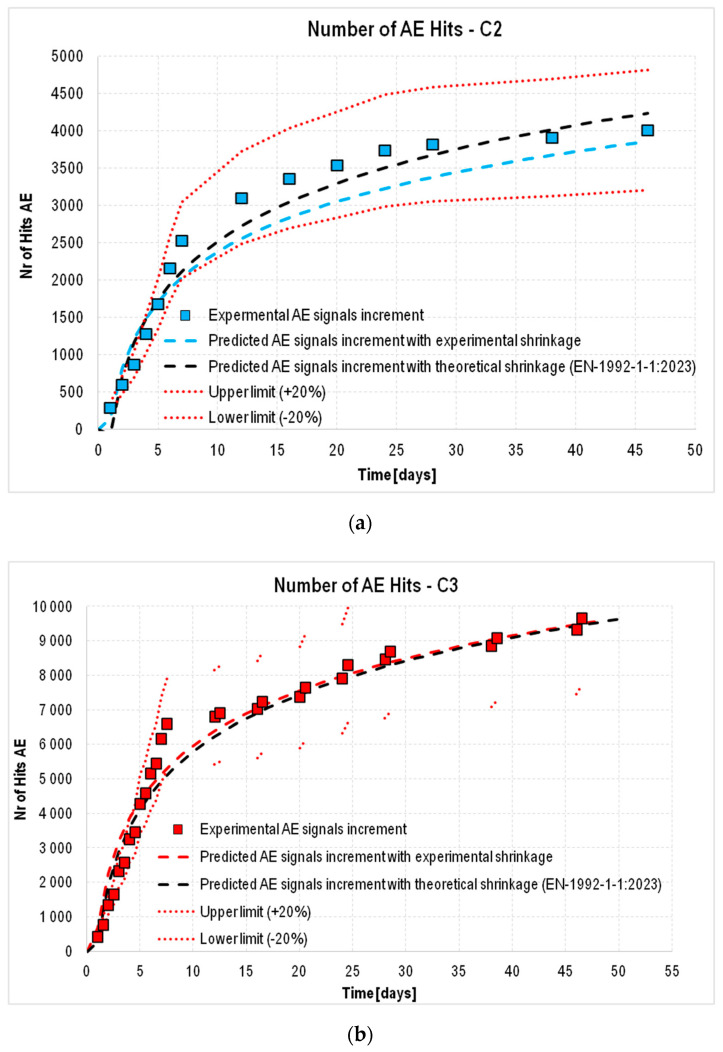
Increase in AE signals recorded during the test accompanied by the theoretical increase estimated from experiments (blue and red dashed line) or calculated from EN 1992-1-1 (black dashed line) shrinkage strain: (**a**) C2 concrete, (**b**) C3 concrete.

Analyzing the results, it was found that using only the coefficients α and β obtained for C1 concrete to determine the theoretical growth of AE signals based on the trend line of the actual shrinkage strain of the C2 and C3 concretes was not sufficient. This fact is illustrated in Figure 16a,b by the black dashed line, which represents the theoretical trend line of the increase in the number of AE signals based on the actual shrinkage strain of the concretes tested. In these cases, due to additional factors—lack of curing (C2) and cyclically varying temperature (C3)—an additional correction factor *γ* was required, resulting in Equation (4):

(4) $$y=\left[\alpha \cdot A\cdot \ln\left(x\right)+\beta \cdot B\right]\cdot \gamma$$

Implementing the coefficient resulted in a better fit of the theoretical values (predicted by standard [24] to the experimental ones for the C2 and C3 concretes, within the accepted error limits of ±20%.

The next step of the analysis was to verify whether the number of acoustic signals could be predicted not from experimental, but from theoretical values of shrinkage strain. In this case, the same procedure as before was used, taking C1 concrete as a reference. This is because a very high correlation was obtained between the experimental results and the predicted results for this concrete. For C2 and C3 concretes, insufficient agreement was obtained between the predicted and experimental strain, probably due to the lack of curing and cyclically variable temperatures. Therefore, an additional coefficient η was determined, which adjusted the indicated differences in the strain values and made it possible to obtain a satisfactory convergence of the trend lines of experimental and predicted strain. As a result, Equation (4) was modified to the form (5):

(5) $$y=\left[\alpha \cdot A\cdot \ln\left(x\right)+\beta \cdot B\right]\cdot \eta \cdot \gamma$$

The correction factors α, β, *γ,* and η used in the analysis are shown in Table 5.

The results obtained based on the coefficients derived (Table 5) are presented in Figure 16a for C2 and Figure 16b for C3.

Based on the analysis performed, it was shown that it is possible to predict the increase in the number of AE signals (destructive processes) based on the shrinkage strain using both the experimental values of the shrinkage strain and the values determined from the model used in the European standard [24].

To sum up the analysis steps, in order to estimate the increase in the number of acoustic emission signals based on the shrinkage strain calculated according to the formulas contained in EN 1992-1-1, according to Equation (5), the following should be done:

- Apply coefficients α and β (Table 5), which allow for the transition from the logarithmic trend line of the increase in contractile strain to the logarithmic trend line of the increase in AE signals.
- In the case of concrete without curing, an additional factor, η (Table 5), should be applied.
- If the concrete has been exposed to variable temperatures, factor *γ* (Table 5) must also be taken into account.

As the analysis showed, in addition to the coefficients, co-factors are needed due to the fact that the shrinkage strain calculated according to the formulas contained in EN 1992-1-1 are underestimated in the case of non-cured concrete and of non-cured concrete with variable temperature, as shown in Figure 9b,c and Figure 10b,c.

It should also be noted here that the conducted analysis and the results concern primarily C30/37 class concrete made on the basis of Portland cement subjected to the external factors presented in the analysis. Further research planned by the authors will aim to verify and correct the presented approach in the context of other classes of concrete, cements, and the use of, for example, dispersed reinforcement.

## 4. Discussion

In this study, the dependence between shrinkage strain and microcracks recorded by the acoustic emission method (IADP) formed in concrete matured at a constant temperature of 22 °C, cured in water, and dried in air, as proposed in [52], was confirmed for the tested concrete. The strain estimated according to the Eurocode 1992-1-1:2008 standard [23] analyzed in [52] for concrete without prior curing indicated significant differences from experimental results. In the experimental deformation study, the results were compared with the newer version of Eurocode 1992-1-1:2023, again confirming the differences from experimental test results for untreated concrete after removal from the molds.

There was a correlation between the experimental results of the strain obtained for C1 (subjected to curing) and those estimated according to the standard model proposed in the PN-EN-1992-1-1:2024 standard [29]. At the same time, discrepancies were observed between the experimental results and the strain estimated according to the [24] standard in the case of concretes with incorrect curing or lack of curing and when the concrete was additionally subjected to a cyclically variable temperature, alternately heated and cooled. In this case, the values calculated according to the standard approach were underestimated by up to 60% (in 46 days of hardening). The authors introduced coefficients to correct for the mentioned discrepancies.

Determining the coefficients α and β (correlation coefficients) from the results for C1 concrete, along with the determination of additional correction coefficients *γ*, was conducted to include in the analysis such factors as lack of curing and cyclically varying ambient temperature. The results were within ±20% error with respect to the experimental values, based on both experimental and theoretical values of shrinkage strain.

The results presented in this paper confirmed the hypothesis that shrinkage strain, recorded experimentally in concrete and also estimated according to the most recent version of the Eurocode 2 (EN-1992-1-1:2023) standard [24], correlate with destructive processes. Above that, it was observed that it is necessary to develop a database of results to refine the values of correlating coefficients for concrete with other parameters than those studied in the article, since the research carried out in the article concerned concretes on basalt aggregate with blast furnace cement.

The authors of this paper tested young concrete, among other things, in a non-standard range of temperature and humidity, which was designed to expose specimens to an adverse environment and as a result to see what destructive processes can occur in such situations. The results of the tests may be relevant when concreting in cold conditions, when water can freeze before the concrete reaches adequate strength, or with large temperature amplitudes. In the literature, we can find studies such as [57], where concrete was subjected to freeze–thaw cycles, with different temperature ranges adopted in the work, the number of cycles, or the minimum age of concrete at the time of testing (minimum 14 days or 28 days). However, these studies only analyzed the AE signals without measuring the shrinkage strain, which was one of the main objectives of this paper, i.e., to show the relationship between the two phenomena. Furthermore, it should be noted that in future research, tests based on the analysis of concrete frost resistance should not be excluded. However, it should be taken into account that standard tests related to the frost resistance of concrete do not assume the measurement of shrinkage, nor are they aimed at testing young concrete in the first few days after removing the samples from the molds, when the greatest shrinkage deformations occur.

## 5. Conclusions

Based on the tests and analyses performed, the following conclusions can be drawn.

1. The EN 1992-1-1:2023 [24] standard (implemented in Poland as PN-EN 1992-1-1:2024) describes the strain of samples subjected to curing with water well.
2. In the case of samples without water curing, for both constant and variable temperature, it is necessary to use the coefficient 1.65, which corrects the values estimated according to the Eurocode 2 standard.
3. Shrinkage strain is accompanied by destructive processes: microcracks in the cement paste, development of internal cracks, and the formation of microcracks on the concrete surface, which can be identified using AE (signal classes 1, 2, and 3).
4. The number and energy of destructive processes is a linear function of the strain for samples with and without curing in water.
5. The total number of destructive processes (AE signals) can be determined as a function of strain, both experimentally measured and standard-predicted (taking into account correction factors for samples without curing in water).
6. For samples without water curing, class 3 signals should be recorded, which indicate processes related to the formation of microcracks on the surface that may affect the durability of the elements.

The analysis presented in this paper regarding the correlation between the increase in shrinkage strain and the increase in acoustic emission signals is of a preliminary nature and was intended to present the observed phenomena.

According to the results presented and description of the tested samples, the results refer only to the concrete based on blast furnace cement subjected to the external factors presented in the paper.

As a result, more research is planned to verify the results presented and to extend the scope of applicability of the dependences presented in the work.

The scope of the planned tests includes, among others: different classes of concrete, different cements including low-emission cements, curing conditions, and dispersed reinforcement. However, due to the wide scope of the planned tests, compiling all of the results and their analysis will be a long-term process.

## Figures and Tables

**Table 1:** Performance properties of CEM III/A 42.5N-LH/HSR/NA blast furnace cement.

Performance Properties	Values
Components:	
- Portland cement clinker (K)	35 ÷ 50%
- granulated blast furnace slag (S)	50 ÷ 65%
- secondary components	0 ÷ 5%
Compressive strength:	
- strength class	42.5 N
- after 2 days	Above or equal to 10 MPa
- after 28 days	Between 42.5 MPa and 62.5 MPa
Bonding time	Above or equal to 60 min (beginning)
Undissolved residue	Below or equal to 5.0%
Loss of roasting	Below or equal to 5.0%
Stability of volume:	
- expansion	Below or equal to 10 mm
- sulfur trioxide content	Below or equal to 4.0%
Chloride content	Below or equal to 0.10%
Total alkali content	Below or equal to 1.10%
Heat of hydration	Below or equal to 270 J/g

**Table 2:** Category or declared values for selected parameters of the basalt aggregate.

Parameter	Basalt Aggregate 2/8 [54]	Basalt Aggregate 8/16 [55]
Specific gravity [Mg/m^3^]	3.18	3.17
Water absorption [%]	0.9	0.7
Dust content [%]	1.5	1.5
Frost resistance in the presence of salt [%]	1	1
Resistance to crushing	LA_15_	LA_15_
Resistance to abrasion	M_DE_10	M_DE_20
Resistance to polishing	PSV_50_	PSV_50_
Resistance to surface abrasion	AAV_10_	AAV_10_
Frost resistance	F_1_	F_1_
Reactivity of alkaline	Non-reactive	Non-reactive

**Table 3:** Performance properties of concrete mixtures.

Symbol	Density g/dm^3^	Cone Drop Test [cm]	Air Content [%]	Consistency
C1	2523	2.8	1.7	plastic
C2	2509	2.0	1.8	plastic
C3	2517	1.5	1.2	plastic

**Table 4:** Average unit energy of AE signals and corresponding destructive processes of classes 1, 2, and 3 recorded in concrete of the C3 series.

Concrete Series	Class 1	Class 2	Class 3
C3	0.28	21.01	124.91

**Table 5:** List of coefficients applied depending on the concrete series.

Concrete Series	Conditions	α	β	η	*γ*
C1	Cured, temp. = constant	33,280	11,775	1.00	1.00
C2	Without cure, temp. = constant			1.65	
C3	Without cure, temp. = cyclic				0.52

## Data Availability

The original contributions presented in this study are included in the article. Further inquiries can be directed to the corresponding authors.
